# GROP: A genomic information repository for oilplants

**DOI:** 10.3389/fpls.2022.1023938

**Published:** 2022-10-06

**Authors:** Wenlei Guo, Hongmiao Jin, Junhao Chen, Jianqin Huang, Dingwei Zheng, Zhitao Cheng, Xinyao Liu, Zhengfu Yang, Fei Chen, Kean-Jin Lim, Zhengjia Wang

**Affiliations:** ^1^ State Key Laboratory of Subtropical Silviculture, College of Forestry and Biotechnology, Zhejiang A&F University, Hangzhou, China; ^2^ Department of Biology, Saint Louis University, St. Louis, MO, United States; ^3^ College of Tropical Crops, Sanya Nanfan Research Institute, Hainan University, Haikou, China; ^4^ Hainan Yazhou Bay Seed Laboratory, Sanya Nanfan Research Institute, Hainan University, Sanya, China

**Keywords:** genomic data, oil plants, bioinformatics, information repository, transcriptomic data

## Abstract

Biomass energy is an essential component of the agriculture economy and represents an important and particularly significant renewable energy source in the fight against fossil fuel depletion and global warming. The recognition that many plants naturally synthesize hydrocarbons makes these oil plants indispensable resources for biomass energy, and the advancement of next-generation sequencing technology in recent years has now made available mountains of data on plants that synthesize oil. We have utilized a combination of bioinformatic protocols to acquire key information from this massive amount of genomic data and to assemble it into an oil plant genomic information repository, built through website technology, including Django, Bootstrap, and echarts, to create the Genomic Information Repository for Oil Plants (GROP) portal (http://grop.site/) for genomics research on oil plants. The current version of GROP integrates the coding sequences, protein sequences, genome structure, functional annotation information, and other information from 18 species, 22 genome assemblies, and 46 transcriptomes. GROP also provides BLAST, genome browser, functional enrichment, and search tools. The integration of the massive amounts of oil plant genomic data with key bioinformatics tools in a database with a user-friendly interface allows GROP to serve as a central information repository to facilitate studies on oil plants by researchers worldwide.

## Introduction

The advancement of human society has required enormous amounts of energy in many forms, beginning with firewood and expanding to coal and then to petroleum, natural gas, and kerogen shale. Fossil fuels began to occupy a crucial position when civilization became industrialized with the introduction of steam power. Now, however, the excessive dependence on fossil fuels has become a potential threat to civilization. Among the 125 questions about exploration and discovery published in *Science* (Levine et al., 2021), three are related to energy: (1) Can we stop global climate change? (2) Where do we put all the excess carbon dioxide? and (3) Could we live in a fossil fuel–free world? The key answer to all three of these questions is probably biomass energy.

One significant contributor to biomass energy is oil plants—plants that naturally synthesize hydrocarbons, predominantly lipids, *in vivo.* The oil plants comprise a large group of herbs, shrubs, and trees, with commercial species that include oil palm (*Elaeis guineensis*), oilseed rape (*Brassica napus*), peanut (*Arachis hypogaea*), and soybean (*Glycine max*). In these plants, the lipids are mainly stocked in their seeds, although other species store hydrocarbons in their leaves, fruits, or stems. Plant lipids can be classified into five broad categories: (1) fatty acids with 16 to 18 carbons; (2) very long chain fatty acids with over 18 carbons; (3) polyunsaturated fatty acids; (4) hydroxy fatty acids; and (5) wax esters. These valuable lipids play vital roles in the food, paint, lubricant, feed, and medical industries due to their unique chemical properties (Bates et al., 2013; Belayneh et al., 2018). Some of these plant lipids are now recognized as having great medicinal value. For instance, omega-3 fatty acids, such as eicosapentaenoic acid (EPA) and docosahexaenoic acid (DHA), are essential in the human diet, as they cannot be synthesized *in vivo*, and they are now known to reduce heart attack risk(Ruxton et al., 2004) and to be effective in treating neurodegenerative and neurological disorders(Dyall, 2015), cancer(Moloudizargari et al., 2018), fetal development disorders (Dunstan et al., 2008), and cardiovascular disease(Bouwens et al., 2009). However, oil plants are now increasingly being recognized for their biomass value and development potential.

The growing desire to capitalize on the significant industrial and ecological value of oil plants has led to a multitude of scientific projects aimed at improving the yield and quality of these plants. Thanks to the rapid development of sequencing technology, an enormous collection of genome sequencing and transcriptomic data has been generated for many oil plants in recent years. The first wild olive (*Olea europaea*) genome (≈1.48G) was completed and published in 2017, and two Oleaceae-specific paleopolyploidization events were identified, leading to expansion and new functionalization of several gene families (*ACPTE*, *EAR*, *FAD2*, and *FAD2*) involved in lipid synthesis (Unver et al., 2017). For oil plants used in cosmetics and as lubricants, researchers have utilized a combination of multiple sequencing techniques, such as PacBio, Illumina, and Hi-C, to assemble a high-quality chromosome-level jojoba (*Simmondsia chinensis*) genome (≈887Mb, 2n=26) (Sturtevant et al., 2020). Oilseed rape (*Brassica napus*), the plant that produces the well-known canola oil that accounts for approximately 13–16% of all globally consumed vegetable oils (Wang et al., 2018), was “recently” formed about 7500 years ago by natural hybridization and polyploidization (Chalhoub et al., 2014), so the complex genome assembly of different accessions has been sequenced and improved many times (Chalhoub et al., 2014; Bayer et al., 2017; Sun et al., 2017; Zou et al., 2019; Song et al., 2020; Chen et al., 2021).

These advances have revealed important information about stress responses, domestication, and lipid synthesis in oil plants at the genomic level. The rapid accumulation of genomic data has also accelerated the process of plant molecular breeding by identifying and locating precise gene targets. Thus, constructing a database that can integrate, share, and visualize genomic and transcriptomic data for oil plants has become a necessity for the research community. We have addressed this need by constructing a public repository, the Genomic Repository of Oil Plants (GROP, www.grop.site), that stores and shares the genomic and transcriptomic data of oil plants.

GROP is the first digital resource library to store a range of oil plant genomic data, including genes, genome sequences, genome features, gene annotations, and transcriptome profiles. GROP also provides a batch of search tools and data visualization functions, including gene, gene family, transcription factor, protein kinase, and keyword searches, which allow users to retrieve relevant information quickly from the large collection of genomic data. A Basic Local Alignment Search Tool (BLAST) server has been deployed in GROP to integrate genomic, gene nucleotide, and protein sequences. A genome browser was also embedded in GROP for the integrative visualization of genomic sequences, annotation data, and functional genomic data. GROP also includes tools for Gene Ontology (GO) enrichment, pathway enrichment, and expression visualization that allow users to perform functional analyses of their gene sets. We expect that GROP will serve as an efficient genomic data center for the research community interested in oil plants. We plan to continuously update GROP to include newly generated genomic data.

## Material and methods

### Genomic and transcriptome data resource acquisition

Genomic data includes genome sequences, coding sequences (CDS), protein sequences (PEP), and genome structure annotation files (GFF) that are essential for in-depth exploration of oil production by plants. The original genomic data sources in GROP are composed of two parts, with one part coming from major public bioinformatics databases and the second part being a new version of the pecan (*Carya illinoinensis*) genome assembly contributed by our research team. The raw sequencing data of the 46 oil plant transcriptomes in the database were downloaded from the NCBI Sequence Read Archive (SRA) database, which comprises RNA-seq from different tissues at different developmental stages and from plants in different environments. The RNA-seq data were downloaded using the SRA toolkit. All data can be traced in the database (**Supplementary Table S1**).

### Gene annotation

Gene function annotation compares a gene sequence or protein sequence with bioinformatics databases to predict the function of the gene. In GROP, the latest InterPro (Finn et al., 2016) protein resource package (v86.0) and Panther classification data were collected. We then used InterProScan v5.52 software to perform functional predictions against protein sequences with 30 threads and a GO term output mode. These annotation results provide comprehensive prospects for potential functions. The KEGG online analysis service BlastKOALA (Kanehisa et al., 2016) was utilized to identify vital genes in the KEGG pathways.

Gene family or protein domain classification was conducted using Pfam (El-Gebali et al., 2019). We first obtained the conserved domain feature resources included in the Pfam database, and we then used the HMMER software (Finn et al., 2011) to search all the oil plant protein sequences against the HMM seeds. Transcriptional factors and protein kinases were identified using the iTAK software (Zheng et al., 2016) with the default parameters.

### Expression profile calculations

For the 46 oil plant transcriptome datasets, a standard and optimized pipeline was set up for automated calculations: (1) fastq-dump command with split-3 was executed to transform SRA data to the fastq format data using the SRA toolkit; (2) fastp (Chen et al., 2018) was used for quality controlling, filtering, adapter trimming, and per-read quality pruning; (3) a genome index was built and the raw sequencing data mapped to the corresponding genome to obtain a BAM file using the STAR software (Dobin et al., 2013); and (4) the gene expression value was calculated and normalized to FPKM (fragments per kilobase of exon per million mapped fragments) and joint different samples to a complete matrix using the RSEM software (Li et al., 2009). These final expression profiles were ultimately uploaded to the cloud server.

### BLAST, genome browser, and FTP server implementation

We provide clear and easy use of similar sequence search services for oil plant researchers by deploying an online BLAST service based on the coding, protein, and genome sequences of 22 oil plant genome assemblies. This BLAST service is set up using SequenceServer (Priyam et al., 2019) and optimized with the jstree module, which splits the sequence library into three parts (coding, protein, and genome sequences) and classifies different assemblies that belong to the same species into one node.

The genome browser is deployed on the Linux server so that users can access the genomic information intuitively and interactively using multiple web browsers (Chrome, Firefox, IE, Safari, etc.). We configured an extendable genome browser using Jbrowser (Buels et al., 2016) to integrate genomic information about sequences, gene structure, RNA-seq, mutation sites, etc.

We have provided a rapid data acquisition channel for worldwide researchers by setting up a File Transfer Protocol (FTP) download site utilizing the vsftp technique. In this case, complete genomic sequences, genome structure annotation data, multifold functional annotation data, and 46 RNA-seq expression matrices have been cached on the FTP site.

### Data model and website implementation

**Figure 1:**
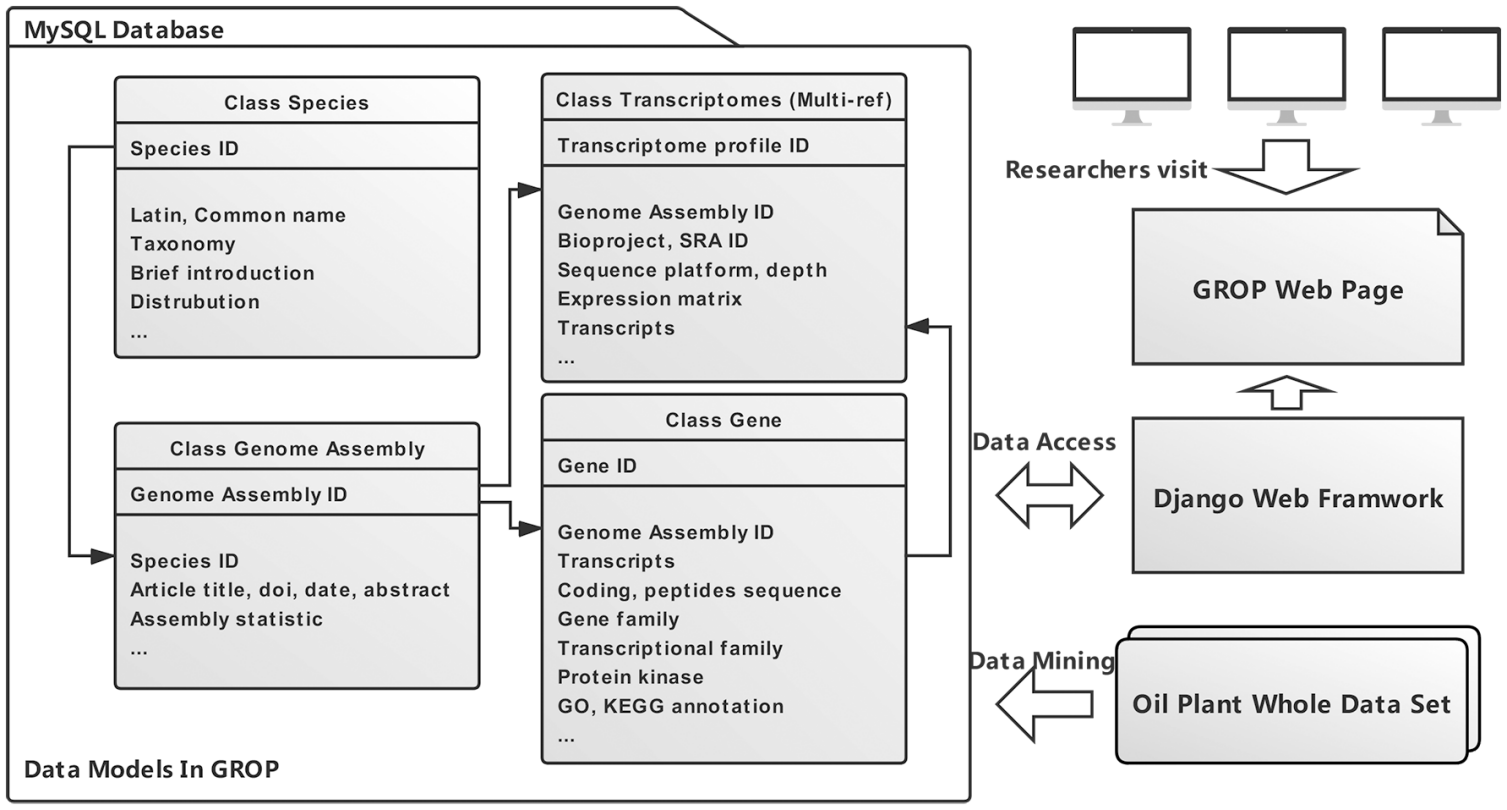
An overview of the GROP complete architecture. In general, four data models (species, genome assembly, transcriptome profile, and gene) were built through data mining from an oil plant whole dataset. Researchers are able to visit GROP *via* the Django web framework.

The data structure and relationship of the four most significant data models (Species, Genome assembly, Transcriptome profile, Gene) are shown in **Figure 1**. These four data models are instrumental in the data retrieval and assay procedures within the whole dataset (**Table 1**). In general, GROP was established using a series of modern website development techniques, including HTML, CSS, Bootstrap, MySQL, and Django. Django was the key element connecting the front webpage and genomic information, and it facilitates the development process. The Django framework also allows us to decorate a data management system that will facilitate future data updates. The whole GROP project has been deployed on a cloud server using a mixture pipeline of Gunicorn and Nginx, as we described previously (Guo et al., 2020). Ultimately, a user-friendly and usage-flexible public genomic platform has been built for the oil plant scientific community.

**Table 1:** Data statictis of dataset in genomics respository of oil plant.

Data model	Item count
Species	18
Genome assembly	22
Transciptome profile	46
Gene	413,509
Gene family	586,165
Transcriptional factor	33,485
Protein kinase	16,405
GO annotation	3,649,008
KEGG annotation	409,358

## Results

### The GROP homepage

**Figure 2:**
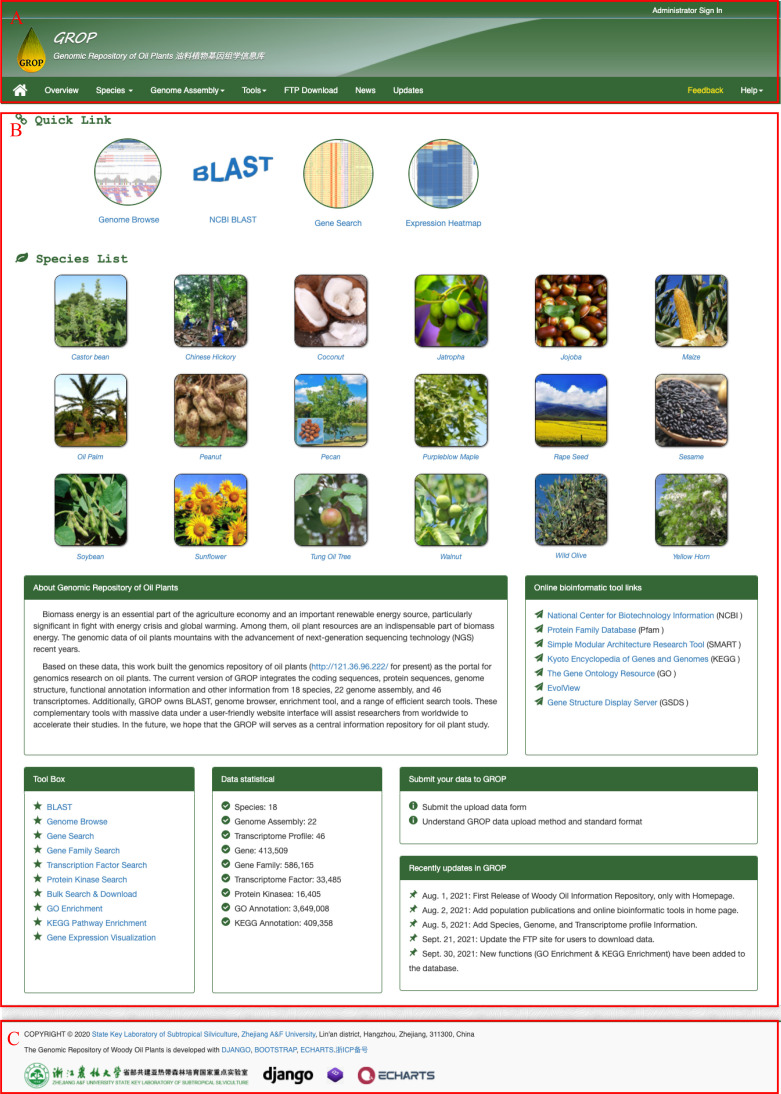
Homepage of the GROP database. **(A)** Navigation bar of GROP, including the species introduction, tool links, etc. **(B)** Main content, including a quick link for the four most useful tools, species list, repository introduction, etc. **(C)** Website footer of GROP.

The homepage of the Genomic Repository of Oil Plants (**Figure 2**) consists of three parts, from top to bottom: a navigation bar, the main content, and footer content. The homepage also offers links to several important bioinformatic resources and services. The navigation bar includes multiple dropdown menus that link to important information and online tools, such as “Species,” “Genome assembly,” and “Tools.” The “Species” dropdown menu in the navigation bar (**Figure 2A**) lists the 18 species currently included in GROP. Clicking on a species name redirects users to its description page. The “Genome assembly” dropdown menu currently lists 22 genome assemblies for 18 oil plants and links to a new page that includes the statistical information of the genome assembly and the abstract of its publication. The “Tool” contains links to all the tools, which are described in detail in the sections below. GROP also allows users to download all raw data with an FTP protocol, and the link is also included in the navigation bar. The main content (**Figure 2B**) is divided into three sections, from top to bottom: the top section contains the four most used tools in GROP; the middle section shows pictures and links to the 18 oil plants in the GROP; and the bottom section includes an introduction, links, toolbox, and data statistics and recent updates. At the bottom of the homepage, the footer section (**Figure 2C**) shows the logos of the techniques used for the development of GROP and provides the author contact information.

### BLAST and genome browser for genomic data

**Figure 3:**
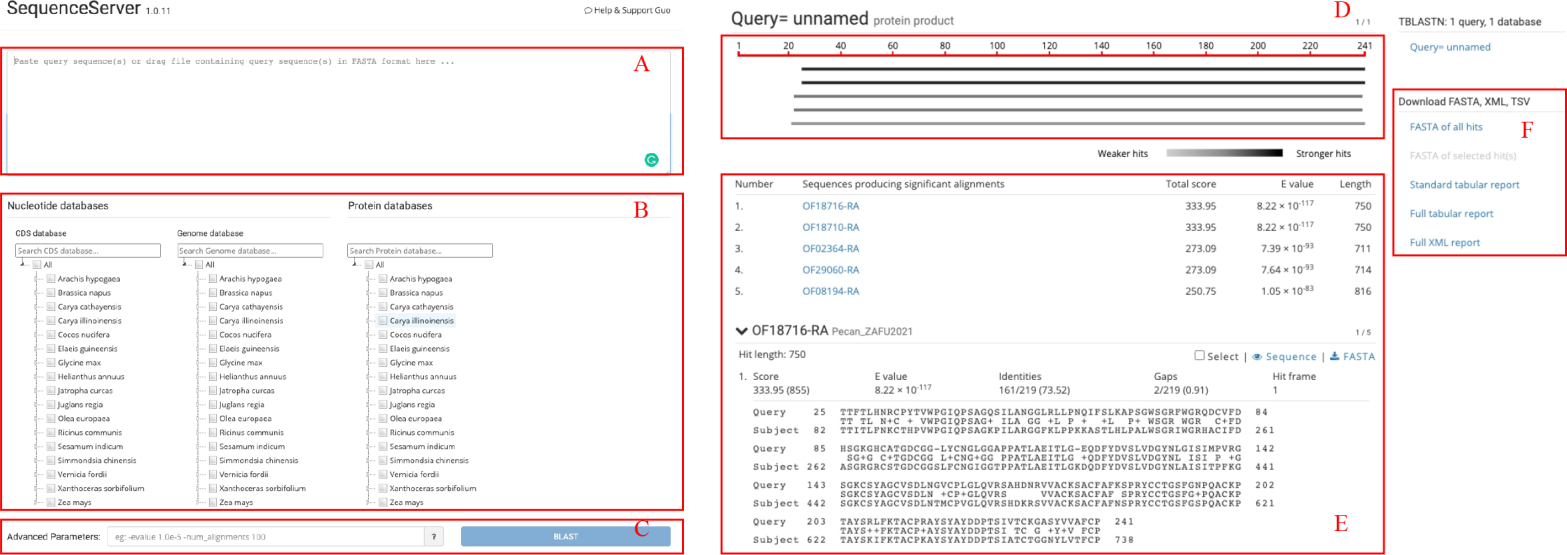
BLAST interface of GROP. **(A)** Query sequence input box for the BLAST program. **(B)** Search databases containing nucleotide and protein sequences. **(C)** BLAST advanced parameter input box. **(D)** Visualized BLAST alignment map. **(E)** Detailed BLAST result information. **(F)** BLAST results for download.

GROP provides a BLAST tool for sequence similarity searches. The gray box at the top of the BLAST interface (**Figure 3A**) is the input box for the query sequences. In the middle of the page, the user can select a target sequence database that includes coding, protein, and genomic sequences for each species (**Figure 3B**). The optional advanced parameters, such as the E-value threshold and the number of alignments to be shown, can be chosen in the input box at the bottom of the page (**Figure 3C**). The returned results are divided into three parts: the top left part is a graphical representation of the Blast hits found (**Figure 3D**) and provides a quick overview of the query sequence and the resulting hit sequences. The bottom left part shows the BLAST table and alignments of the BLAST results (**Figure 3E**). The download links are provided in the right half (**Figure 3F**), allowing users to download BLAST results in either the tab-separated or XML formats.

**Figure 4:**
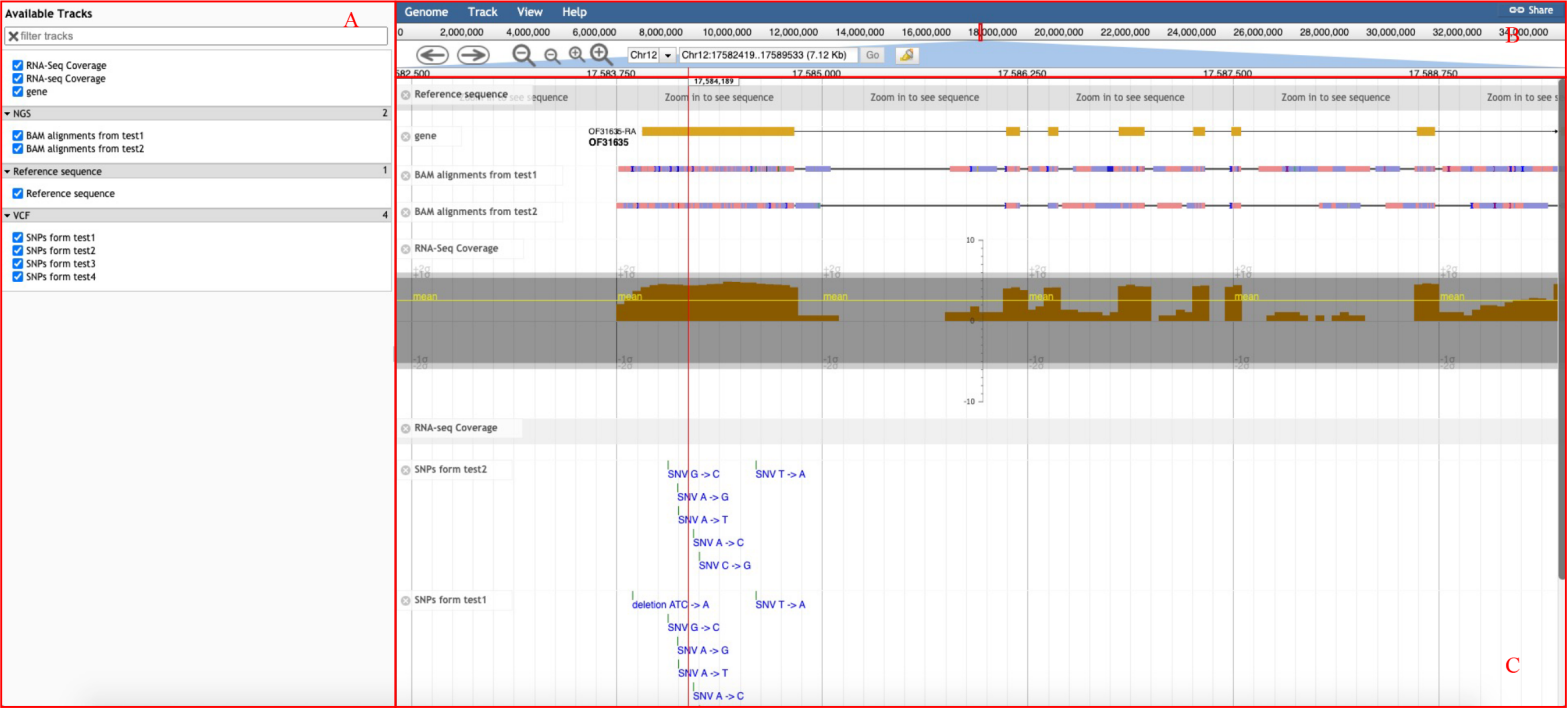
Genome browser interface of GROP. **(A)** Selection bar for genomic information in the browser. **(B)** Navigation bar for chromosome position, zoom in and out. **(C)** Visualization interface for selected genomic information.

The basic interface of the genome browser (**Figure 4**) provided by GROP includes three parts: the selection bar on the left (**Figure 4A**), the navigation bar on the right (**Figure 4B**), and the main display of the Genome Browser (**Figure 4C**). Users can select an oil plant species from a list in the navigation bar. Users can also upload various types of genomic annotation information to be displayed in the main panel. Available tracks also provide users with annotated information of the gene structure and other desired genomic information. For example, BAM files can be uploaded to the genome browser for visualization of aligned reads to the reference genome and to detect base mismatches, insertions, deletions, and other variation information. With these functions, users can visually analyze their own genomic data of interest.

### Single gene search

The single gene search is one of the most important search tools in GROP. The gene search tool can be implemented by entering a gene identifier, which can be obtained in various ways (BLAST, JBrowse, etc.). The gene search returns the source organism, gene structure, sequence length, gene family, coding, and protein sequences, and expression level. The functional annotation information from various bioinformatic databases, such as Amigo, CDD, Gene3D, InterProScan, MobiDBLite, Pfam, and Swiss-prot, is also provided in the gene research results.

### Bulk search and download

GROP provides multiple search methods to find clusters of conserved genes, including gene families, transcription factors, and protein kinases. Entering the Pfam ID of a gene family and genome assembly into the gene family search function will return a list of genes. Several methods to manipulate sequences in bulk are also supported at the top of the gene list page. GROP also provides a keyword search tool, based on an SQL fuzzy search for the entire database and including gene coding, protein, and genome sequences, as well as InterProScan, KEGG metabolic pathway, and Pfam annotation data.

An FTP download site was built with the complete data resources for oil plants. This download site stores the nucleic acid sequences of coding proteins, protein sequences, genome sequences, genome structural features files for each genome, functional annotation information about protein domains, GO terms, and KEGG metabolic terms. We have also integrated the gene expression data in the FTP site. Through the site, researchers can download the entire dataset in the repository.

### Enrichment tool for GO and pathway terms

**Figure 5:**
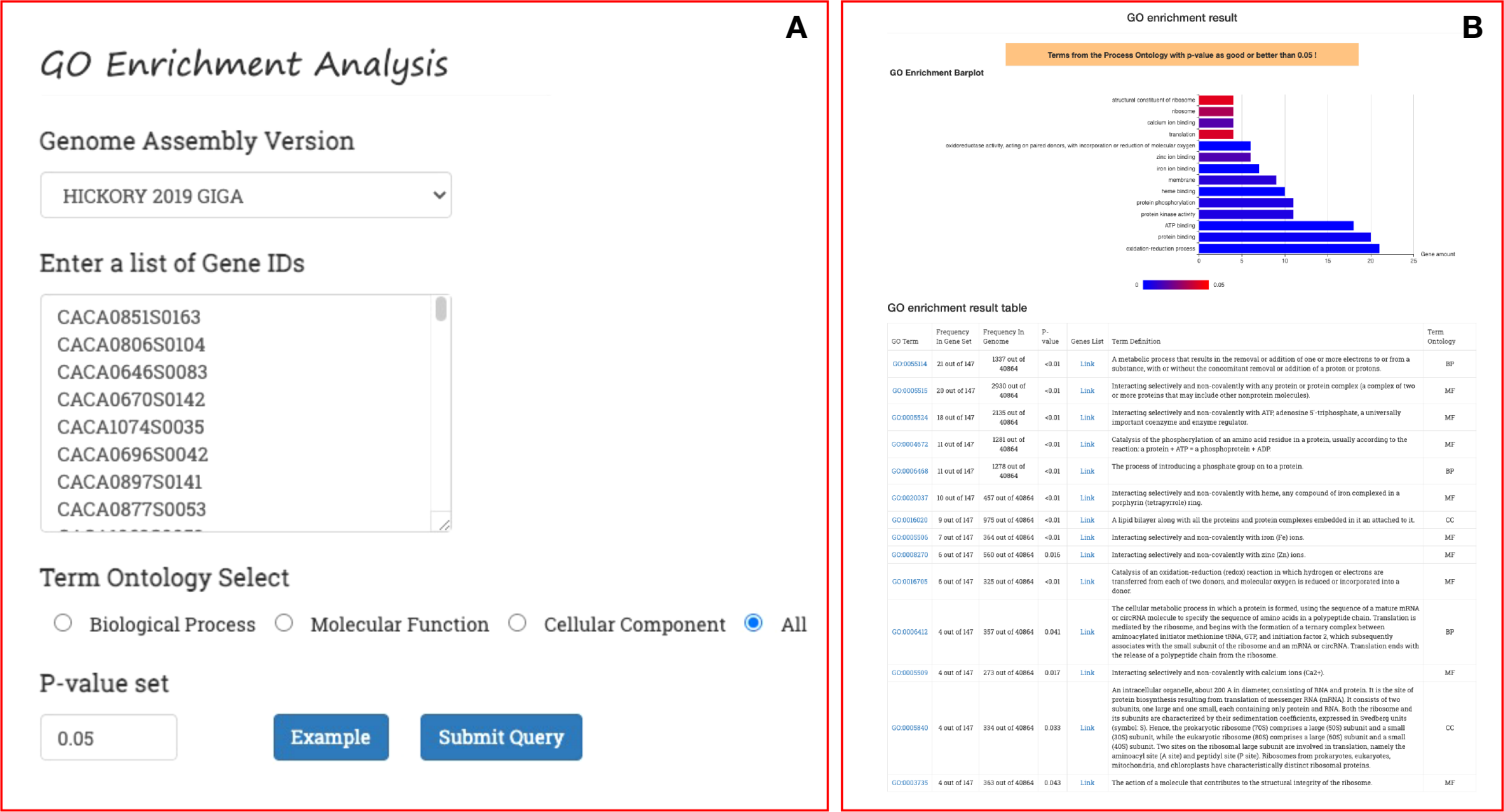
GO and KEGG enrichment analysis process in GROP. **(A)** Submission form for genome version, gene set, ontology term, and p-value. **(B)** Returned enrichment result, including a histogram and a summary table.

On the GROP site, GO enrichment analysis can be performed with a simple workflow: select the genome version of the oil plant; select an ontology category (Biological Process, Cellular Component, Molecular Function, or All); and provide a list of gene IDs and the threshold p-value (**Figure 5A**). The result of the enrichment analysis returned by GROP is a bar plot of the enrichment analysis, where each bar represents an enriched GO term, the length of the bar indicates the number of genes included in the GO term, and the shade of the bar indicates the p-value, with a color closer to red indicating a smaller p-value with higher confidence (**Figure 5B**). A table of the results of the enrichment analysis, presented beneath the bar graph, lists each enriched GO ID and the gene frequency, p-value, link to the gene list, GO function description, and annotation link. With this tool, researchers can conveniently perform GO enrichment and KEGG enrichment analysis on a specific list of genes in the 18 oil plants, thereby eliminating the need to use the R package or other programs.

### Expression visualizer

The heatmap is a popular visualization of gene expression data that shows the expression levels of multiple genes or transcripts in different environments, developmental stages, or tissues. Researchers can easily comprehend the gene expression pattern under different backgrounds.

**Figure 6:**
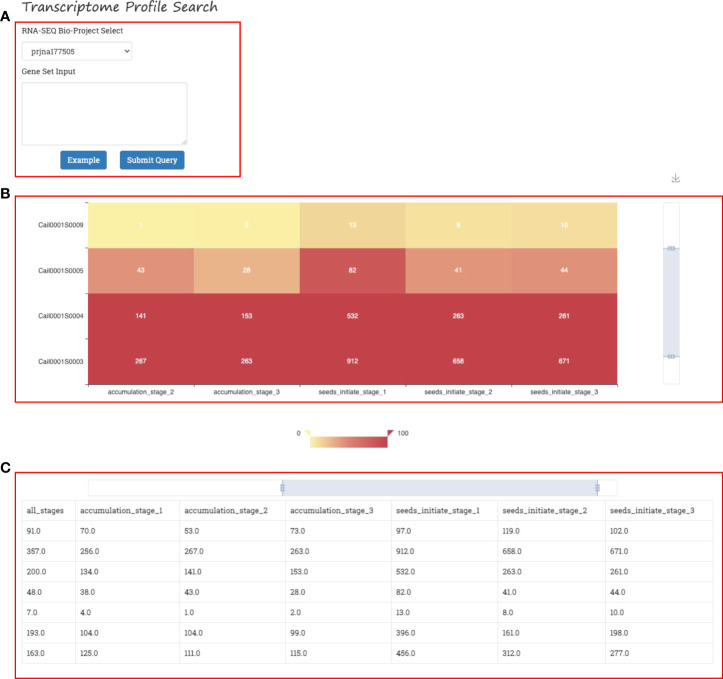
Gene expression visualizer in GROP. **(A)** Input panel of the transcriptome version and gene set. **(B)** Gene expression heatmap for different gene sets, environments, and developmental stages. **(C)** Gene expression data matrix.

A gene expression heatmap is generated in the Expression Visualizer of GROP when the users select a RNA-seq Bioproject from the dropdown list and then provide gene IDs of the query. (**Figure 6A**). The gene expression visualizer returns an expression heatmap of the input genes of the corresponding transcriptome (**Figure 6B**). The horizontal axis and vertical axes of the heatmap represent different genes and different samples in the transcriptome, respectively. The heatmap is available in different scales and can be downloaded. Gene expression data are also provided as a data matrix at the bottom of the web page for further analysis (**Figure 6C**).

## Discussion

The agricultural development and utilization of biomass is an important direction in developing renewable energy that can be used to replace traditional fuel oil or coal energy. Oil plants, such as hickory, olive, pecan, soybean, and walnut, are important sources of healthy edible oil as well as important sources of economic income for the food industry and agroforestry. By contrast, jatropha, jojoba, oil palm, and tung oil trees are widely used in manufacturing industries for the production of plastics, lacquers, artificial rubber, printing inks, etc. The oil produced by these oil plants is environmentally friendly and pollution-free and has high economic and environmental value. Therefore, the investigation of germplasm resources, genetics, and molecular regulation of fatty acid synthesis traits has been the main focus of oil crop research.

The rapid development of high-throughput sequencing has allowed the assembly and annotation of the whole genome sequences of many important model plants and field crops; however, the genomes of oil plants are difficult to decipher due to the large proportion of repetitive sequences, high heterozygosity, and large genome size. Nevertheless, in recent years, the reduction in the cost of second-generation sequencing technology and the development of third-generation long-read sequencing has led to breakthroughs and the accumulation of a huge amount of genomic and transcriptomic data for oil plants. Therefore, the current issue is how to effectively analyze, integrate, and share the genomic and transcriptomic data of oil plants in the post-genomic era.

This study is the first to construct a genomic database for oil plants. The work has analyzed and integrated the genomic data for 22 oil plant genomes and 46 transcriptomic datasets using various bioinformatics software, such as HMMER, InterProScan, iTAK, and STAR, and has stored this information in a MySQL database. A user-friendly web platform was also established using Django. The resulting repository provides gene, gene family, transcription factor, protein kinase, and keyword searches, with efficient retrieval. The repository also provides gene ontology (GO) and metabolic pathway (KEGG) enrichment analysis tools to resolve significantly distributed functional or metabolic pathways in gene sets. Researchers are also able to find the expression data for gene sets from 46 transcriptomic gene expression datasets in the Expression Visualizer. The BLAST and the JBrowse browser tools are available in the repository, allowing researchers to search for homologous sequences, browse the location and structure of genes, and view the variation and expression abundance of genes in different species in combination with VCF and BAM files.

Currently, we still anticipate expanding the amount of data and adding omics data, such as metabolomes. Persons interested in further development of GROP are welcomed to share data or to participate in any other kind of collaboration. The construction of the Genomic Repository of Oil Plants will fuel genomics and molecular biology research while enriching our future understanding of oil plants. We believe that GROP will become the data center for oil plant studies, and that efforts in GROP will contribute substantially to oil plant research.

## Data availability statement

The datasets presented in this study can be found in online repositories. The names of the repository/repositories and accession number(s) can be found in the article/**Supplementary Material**.

## Author contributions

ZW, WG, K-JL conceived and designed the research; WG, FC obtained, analyzed the data, organized, and constructed the architecture of website. JH, JC, HJ, DZ, ZC and XL collect the information about genome assembly and transcriptomes. WG, K-JL designed the layout of web pages. WG, HJ wrote, K-JL revised the manuscript. ZW and YZ acquired the funding. All authors contributed to the article and approved the submitted version.

## Funding

This work is supported by the grant from the National Key R&D Program of China (2018YFD1000604); the Zhejiang Agriculture New Variety Breeding Major Science and Technology Special (2021C02066-12); the National Natural Science Foundation of China (32001335).

## Conflict of interest

The authors declare that the research was conducted in the absence of any commercial or financial relationships that could be construed as a potential conflict of interest.

## Publisher’s note

All claims expressed in this article are solely those of the authors and do not necessarily represent those of their affiliated organizations, or those of the publisher, the editors and the reviewers. Any product that may be evaluated in this article, or claim that may be made by its manufacturer, is not guaranteed or endorsed by the publisher.
